# Editorial: Global perspectives on the health inequities in sexual, reproductive, and maternal health post *Roe v. Wade*

**DOI:** 10.3389/fpubh.2024.1488924

**Published:** 2024-09-24

**Authors:** Deborah L. Billings, Ndidiamaka Amutah-Onukagha, Andrea Swartzendruber, Lucy A. Ingram

**Affiliations:** ^1^Arnold School of Public Health, University of South Carolina, Columbia, SC, United States; ^2^School of Medicine, Tufts University, Boston, MA, United States; ^3^College of Public Health, University of Georgia, Athens, GA, United States

**Keywords:** health inequities, sexual health, reproductive health, maternal health, *Roe v. Wade*, *Dobbs v. Jackson Women's Health Organization*

In June 2022, the United States (U.S.) Supreme Court's *Dobbs v. Jackson Women's Health Organization* decision overturned *Roe v. Wade*, thereby eliminating the constitutional right to abortion (1). Authority now resides with individual states to regulate abortion access in the U.S. The impact of the ruling is expected to exacerbate existing health disparities and produce new inequities in sexual, reproductive, and maternal health outcomes, disproportionately affecting those who are already minoritized and living in States where abortion access has been banned or restricted. Observations from countries that have restricted access to abortion over the past 30 years reveal that such laws increase rates of unsafe abortion, which in many instances leads to pregnant people becoming severely ill or dying from preventable causes (2–4). In an era of maternal health crisis for people of color in the U.S. (5, 6) and other disadvantaged populations around the world, eliminating the constitutional right to abortion in the U.S. will have a severe impact on underserved and minoritized groups everywhere (7). This Research Topic of *Frontiers in Public Health* includes ten articles that highlight the global implications of the U.S. Supreme Court decision on sexual and reproductive health.

Several articles illuminated the challenges that the *Dobbs v. Jackson* decision has on reproductive justice. For instance, Montero et al. examined the safety and efficacy of evidence-based abortion care protocols in Chile. They found five types of structural barriers that impede legal voluntary termination of pregnancy (VTP) and conclude that these structural barriers violate reproductive rights and amount to violence against women. Current discourse in the US about the humanity of exceptions to restrictive abortion laws is problematic. This study demonstrates that exceptions do not result in better access to abortion care.

Schott et al. emphasized the importance of ensuring that abortion-related research is conducted ethically and is informed by the social, political, and structural conditions that shape reproductive health inequities. Their discussion underscores that abortion research should be grounded in reproductive justice, human rights, community engagement, and applied ethics.

Roth used a historical framework to examine abortion rights within the U.S., Latin America, and the Caribbean. Roth suggested that reframing restrictions to abortion rights from an issue of individual impacts to a broader public health issue of social and economic justice and human rights will be most effective in advancing reproductive rights.

Lambert et al. examined the anti-abortion rhetoric used in arguments for a 6-week abortion ban in South Carolina. They found that medical disinformation and moral arguments were the most common form of rhetoric used by proponents. A better understanding of the strategies used by anti-abortion supporters can help inform future approaches to abortion and reproductive legislation.

Other authors discussed how the *Dobbs v. Jackson* decision exacerbates existing inequities, most often among marginalized groups. For example, Mann et al. assessed U.S. college students' perspectives on contraception and abortion post-*Dobbs*. Participants were fearful, angry, and concerned about restrictions on reproductive decisions; felt pressured to use certain contraceptive methods [e.g., long-acting reversible contraception (LARC)]; and felt that they would be able to seek an abortion if they desired. The authors concluded *Dobbs* exacerbates the unequal gendered burden of contraception, places undue pressure on young women to use LARCs, diminishes reproductive autonomy, and further illuminates inequities in socioeconomic privilege, particularly given differential perceptions of access to care.

Kheyfets et al. explore the impact of anti-abortion legislation on the Black maternal health crisis in the U.S., highlighting limits to abortion education and training as key factors in worsening health outcomes. The authors also describe the residual impacts of *Dobbs* on access to other reproductive health services. Their approach underscores cascading impacts of restrictive abortion laws on health care delivery and already poor, racialized outcomes in the U.S.

Zhao et al. examined the potential spillover effects of *Dobbs* on non-abortive reproductive care and rights using pre- and post-*Roe* U.S. national clinic data. They concluded that there is early evidence of worsening inequities in non-abortive and reproductive health care differentially impacting socio-economically disadvantaged groups. These insights signal ripple effects regarding how data are collected, how healthcare is funded, how providers are supported, and how comprehensive reproductive health services are delivered that should be considered in policy development.

Andersen et al. studied the impact of Texas Senate Bill 8 on travel to abortion clinics within Texas and out-of-state. Researchers found that travel to abortion clinics in Texas decreased significantly, while travel to out-of-state clinics increased. The study highlights the importance of access to out-of-state abortion services for people in States where abortion is banned or restricted.

Braveman et al. examined California birth records to compare rates of preterm birth among Black immigrants from Africa, Black immigrants from the Caribbean, U.S.-born White women, and U.S.-born Black women who gave birth in California between 2010 and 2021. U.S.-born and Caribbean-born Black women had higher preterm birth rates than U.S.-born white women and African-born Black women. Chronic exposure to stress, such as racism in the U.S., has been linked to this phenomenon. Exposure to discriminatory practices or hostile reproductive environments post-*Dobbs* may have negative impacts on maternal and child health outcomes.

Ujah et al. examined public perceptions and concerns regarding racial and ethnic disparities following the overturn of *Roe v. Wade*. Through sentiment analysis and structural topic modeling, the authors conclude that the ethno-racial concerns following the reversal of *Roe v. Wade* highlight the necessity for ongoing surveillance of racial and ethnic disparities in abortion access post-*Dobbs*. Examining public perceptions regarding legislative changes to health rights may be beneficial in future analysis of policy-related disparities.

The articles in this Research Topic of *Frontiers in Public Health* reveal actual short-term and potential long-term global health inequities to sexual, reproductive, and maternal health produced by the *Dobbs* decision and similar legislation. Exceedingly, authors note compromises to reproductive justice and human rights that suggest calls for advocacy and policies to counter anti-abortion legislation. Devoting a Research Topic to this topic brings vital and robust discourse about reproductive justice and health inequity to the forefront of public health.
